# Lipopolysaccharide-induced CCN1 production enhances interleukin-6 secretion in bronchial epithelial cells

**DOI:** 10.1007/s10565-017-9401-1

**Published:** 2017-06-21

**Authors:** Lin Shi, Nian Dong, Dongxiang Ji, Xiaomin Huang, Zhaojian Ying, Xiangdong Wang, Chengshui Chen

**Affiliations:** 1 Department of Pulmonary and Critical Care Medicine, The First affiliated Hospital, Wenzhou Medical University, Wenzhou, 325000 China; 2 Zhongshan Hospital Institute of Clinical Science, Shanghai Institute of Clinical Bioinformatics, Shanghai Medical College, Fudan University, Shanghai, China; 3 Department of Pulmonary Medicine, Huzhou Central Hospital, Zhejiang, China

**Keywords:** ALI/ARDS, Bronchial epithelial cells, CCN1, IL-6, Inflammation

## Abstract

Acute lung injury/acute respiratory distress syndrome (ALI/ARDS) is a clinical complication caused by primary or secondary lung injury, as well as by systemic inflammation. Researches regarding molecular pathophysiology of ALI/ARDS are immerging with an ultimate aim towards developing prognostic molecular biomarkers and molecule-based therapy. However, the molecular mechanisms concerning ALI/ARDS are still not completely understood. The purpose of the present study was to identify a crucial role of CCN1 in inflammatory microenvironment during ALI/ARDS and focus on a potential communication between CCN1 and interleukin-6 (IL-6) in the airway epithelial cells. Our data illustrated that the expression levels of CCN1 and IL-6 in bronchoalveolar lavage fluid (BALF) in a lipopolysaccharide (LPS)-induced ALI mouse model were significantly elevated and the pulmonary expression of CCN1 was restricted to bronchial epithelial cells. Interestingly, both endogenous and exogenous CCN1 stimulated IL-6 production in vitro. Furthermore, LPS-induced IL-6 production in a bronchial epithelial cell line was blocked by CCN siRNA whereas CCN1 induced by LPS was sensitive to PI3K inhibition. Together, our data indicate a linear signal pathway, LPS-CCN1-IL-6, existing in bronchial epithelial cells after LPS exposure. This finding may represent an additional mechanism and a novel target for development of therapy and biomarker on ALI/ARDS.

**Electronic supplementary material:**

The online version of this article (doi:10.1007/s10565-017-9401-1) contains supplementary material, which is available to authorized users.

## Introduction

Acute lung injury/acute respiratory distress syndrome (ALI/ARDS) is characterized by an excessive inflammatory response and damage of the alveolar epithelial-capillary barrier, associated with different cells and inflammatory mediators (Chen et al. 2016). The over-activated endothelial cells, epithelial cells, and leukocytes could produce and release a large number of inflammatory mediators to initiate and accelerate the secondary inflammatory reactions (Aman et al. 2016). It has been reported that airway epithelial cells can act as the receptor to defend against inflammatory stimuli and antigens, and induce secondary inflammatory responses and systemic reactions in ALI/ARDS through secreting a variety of pro-inflammatory cytokines and chemokines (Wang et al. 2007). Considering the crucial role of airway epithelial cells, better knowledge on how airway epithelial cells act as a communicator during inflammatory cascade reaction potentially provides an innovative aspect to elucidate the complex pathogenesis of ALI/ARDS.

Matricellular proteins play crucial roles in the development of tissue injury repair responses (Kyriakides and Bornstein 2003). CCN1 (also named Cyr61), a cysteine-rich, 38-kD secreted protein, belongs to the CCN family of matricellular proteins that regulate a number of biologic processes such as inflammation and angiogenesis (Kubota and Takigawa 2007; Kular et al. 2011). As an early stress response gene product, CCN1 is expressed in a wide range of cells including airway epithelial cells (Jin et al. 2009; Jin et al. 2005). It can be induced by bacterial and viral infections, or stimuli like TNF-α and IL-1 (Chen and Lau 2009). The secreted CCN1 can interact with integrins and Wnt co-receptors (LRPs) to activate the intracellular signaling pathway in an autocrine and/or paracrine manner (Jun and Lau 2011). Previous studies indicated that CCN1 appeared to act as a new mediator to regulate pro-inflammatory and pro-fibrotic effects, for example, following ischemic kidney injury (Lai et al. 2014) or unilateral ureteral obstruction (Lai et al. 2013). In the lung, CCN1 secretion induced by cigarette smoking augmented IL-8 release from bronchial epithelial cells in the chronic obstructive pulmonary disease (Moon et al. 2013). Additionally, the expression of CCN1 was significantly upregulated in ALI/ARDS (Wallace et al. 2009), and CCN1 overexpression was sufficient to exacerbate lung injury and cause neutrophilic alveolitis and obstructive bronchiolitis in mice (Grazioli et al. 2015; Raissadati et al. 2015). However, the precise biological function of the new inflammatory mediator CCN1 in the setting of ALI/ARDS remains to be elucidated.

IL-6 is a pleiotropic cytokine that participates in acute inflammation as the major inducer of C-reactive protein (Slaats et al. 2016). The concentration of IL-6 was significantly elevated and appeared to correlate with the serum levels of inflammation markers, including TNF-α, IL-1β, IL-8, and IL-18, and predicted the morbidity and mortality of ALI/ARDS (Butt et al. 2016). CCN1 could induce IL-6 expression in inflammatory bowel disease (Choi et al. 2015) and rheumatoid arthritis (Lin et al. 2012). These results identified CCN1 as a novel component of the extracellular matrix (ECM), and can induce IL-6 production during inflammation response. In this study, we hypothesized that the role of CCN1 in inflammatory microenvironment might be involved in the induction of IL-6. In particular, we address the specific cell which can promote CCN1 secretion in ALI/ARDS and explore the potential association and interaction between CCN1 and IL-6. The finding may provide a novel target for development of therapy and biomarker in the ALI/ARDS.

## Materials and methods

### Animals and induction of acute lung injury

Male C57BL/6J mice, weighing 20–25 g (6–8 weeks), were provided by the Animal Facility in the Biomedical Research Center of Zhongshan Hospital, Fudan University. The present study was approved by the Fudan University Ethical Committee for animal experiments. LPS (Product Number L 9143) was originated from *Pseudomonas aeruginosa* (serotype 10, ATCC 27316, phenol-extracted, Sigma-Aldrich, St. Louis, MO, USA). ALI was then induced by the intratracheal instillation of LPS at 4 mg/kg for 4 h. Animals received the same manipulations and volume of PBS as controls. Animal body weight was measured both before the experiment and at termination. After termination, the total number of leukocytes and neutrophils and total levels of protein and IL-6 in BALF were measured. The lung was inflated and fixed with 4% paraformaldehyde under the pressure of 20 cmH_2_O, embedded in paraffin, cut into 5-μm-thick sections, and stained with hematoxylin and eosin. Images were taken with a BX51 microscope DP71 camera (Olympus, Tokyo, Japan).

### Pulmonary endothelial barrier dysfunction and inflammation

Pulmonary inflammation was evaluated by leukocyte migration from the circulation into the alveolar space or local and systematic production of inflammatory mediators. The lungs from animals were lavaged intratracheally with injections of 1 mL PBS, and about 0.8 mL injection was collected in tubes and centrifuged at 1000 rpm and 4 °C for 10 min. The cell pellet was suspended once more in PBS to enable counting of the total leukocyte number. The number of total white blood cells (WBCs) and neutrophils in BALF was counted with Auto Hematology Analyzer (BC-5300 VetTM, Mindray, China). Total protein and IL-6 and CCN1 in BALF were measured with an enhanced BCA Protein Assay Kit (P0010S, Beyotime, China) and murine cytokine-specific Quantikine ELISA Kits (R&D Systems).

### Tissue immunofluorescence and immunohistochemistry analysis

The right lungs of ALI mice were fixed in 4% paraformaldehyde, infiltrated with 30% sucrose/PBS under the pressure of 20 cmH_2_O, embedded in Tissue-Tek OCT compound (Sakura Finetek, Torrance, CA, USA), and fresh-frozen in liquid nitrogen. Five-micrometer and 50-μm-thick sections were stored at −80 °C and then fixed in ice-acetone for 10 min. Then, lung tissues were incubated overnight with mouse monoclonal antibodies against EpCAM, CD31 (Abcam, HK, China, 1:200), or CCN1 (Santa Cruz, CA,1:100), respectively, and then incubated with the corresponding secondary antibodies (Abcam, HK, China, 1:100). The counterstaining was performed with DAPI (4,6′-diamidino-2-phenylindole). Confocal microscopic images were collected with a Leica TCS SL laser scanning confocal microscope (Leica Microsystems, Mannheim, Germany). The left lungs were inflated and fixed with 4% paraformaldehyde under the pressure of 20 cmH_2_O, embedded in paraffin, and then incubated with primary antibody CCN1 and the corresponding secondary antibody.

### Cell lines and reagents

Human bronchial epithelial (16HBE) cells were obtained from Shanghai Institute for Biological Science. Cells were cultured in RPMI 1640 (HyClone, UT, USA) supplemented with 100 U/mL penicillin, 100 mg/mL streptomycin, and 10% heat-inactivated fetal bovine serum (BIO International, Auckland, New Zealand). All cells were maintained at 37 °C in a humidified incubator with 5% carbon dioxide. Human recombinant CCN1 proteins were obtained from PeproTech (Shanghai, China). The rabbit anti-EpCAM as well as rabbit anti-CD31 were purchased from Abcam (HK, China). Mouse anti-CCN1 and horseradish peroxidase-coupled secondary antibodies were obtained from Santa Cruz Biotechnology (Santa Cruz, CA).

### Measurement of gene expression

Quantitative RT-PCR was carried out using real-time PCR with the SYBR Green reporter. Cell cultures were washed in PBS, and total RNA was isolated using a guanidinium isothiocyanate/chloroform-based technique (TRIzol, Invitrogen, USA). RNA was subsequently reverse transcribed to cDNA with the SuperScript First-Strand Synthesis System (Invitrogen, USA). SYBR Green PCR Master Kit was used with the appropriate concentrations (10 nM) of forward and reverse primers in a total volume of 20 μL. Optimization was carried out for each gene-specific primer prior to the experiment to confirm that 10-nM primer concentrations did not produce non-specific primer-dimer amplification signals in no-template control wells. Quantitative RT-PCR was carried out using an ABI 7000 PCR instrument (Eppendorf, Hamburg, Germany) with the two-stage program parameters provided by the manufacturer, as follows: 1 min at 95 °C, and then 40 cycles of 5 s at 95 °C and 30 s at 60 °C. Sequences of the primer sets used for this analysis are as follows: human CCN1, 5′-GCGAGGAGTGGGTCTGTGAC-3′ (forward [F]) and 5′-CTTGTAAAGGGTTGTATAGGATGC-3′(reverse [R]); mouse CCN1, 5′-AAAAGGCAGCTCACTGAAG-3′ (forward [F]) and 5′-GCCGGTATTTCTTGACAC-3′(reverse [R]); IL-6, 5′-GACAGCCACTCACCTCTTCAG-3′ (F) and 5′-CATCCATCTTTTTCAGCCATC-3′(R); mouse glyceraldehyde-3-phosphate dehydrogenase (GAPDH), 5′-AGCAGTCCCGTACACTGGCAAAC-3′ (forward [F]) and 5′-TCTGTGGTGATGTAAATGTCCTCT-3′(reverse [R]); and for human GAPDH, 5′-CCACCCATGGCAAATTCCATGGCA-3′ (F) and 5′-TCTACACGGCAGGTCAGGTCCACC-3′ (R). Specificity of the produced amplification product was confirmed by examination of dissociation reaction plots. Each sample was tested in triplicate with quantitative RT-PCR, and each group had three wells.

### Measurement of protein expression

To measure the expression of CCN1 and the signal pathway induced by LPS, 16HBE cells were cultured in a six-well plate (1 × 10^5^ cells/well) for 24 h and treated with LY294002 at 10, 20, and 30 μM for another 2 h. Then, cells were stimulated with or without LPS at 1 μg/mL for 4 h. Intracellular protein was extracted by RIPA lysis immediately. Protein samples (40 μg) were mixed with a 1× SDS sample buffer, boiled for 5 min, and then separated through 10% SDS-PAGE gels. After electrophoresis, proteins were transferred to PVDF membranes by electrophoretic transfer. Membranes were blocked in 5% dry milk (2 h), rinsed, and incubated with primary antibodies (diluted at 1:500) in TBS thrice (TBST) at 4 °C overnight. Primary antibody was then removed by washing in TBST, and labeled by incubating with 0.1 mg/mL peroxidase-labeled secondary antibodies (against mouse) for 2 h. Following three washes in TBST, bands were visualized by ECL and exposed to X-ray film. Also, we detected the expression of CCNI in ALI mice and in 16HBE that was stimulated with LPS at different concentrations for an indicated time. All results were calculated by Phoretix 1D software.

### Production of IL-6

16HBE cells were cultured in 24 well cell culture microplates at 1 × 10^5^ cells/well for 24 h and then treated with LPS at a concentration of 1 μg/mL for an additional 24 h, respectively, to study LPS-induced IL-6 production. Cells were preincubated with CCN1 small interfering RNA (siRNA) and scramble siRNA before LPS stimulation to explore the role of CCN1 in LPS-induced IL-6 production. Then, we stimulated 16HBE cells with different concentrations of human recombinant CCN1. Each experiment was done in three replicate wells more than three separate experiments for each drug concentration and each time point. The levels of IL-6 in supernatant were measured by ELISA at the absorbance of 450 nm according to the manufacturer’s instructions.

### RNA interference (siRNA) and transfection

CCN1 siRNA and non-specific scramble siRNA were purchased from Shanghai GenePharma Co., Ltd. Transfection of siRNA was performed according to the manufacturer instructions. Briefly, for the transient transfection of siRNA, 16HBE cells were placed in 6- or 12-well plates, optimal 40~60% confluent condition for transfection was determined, and 10–40 pmol siRNA was used for each transfection. And each experiment included controls containing the transfection reagent with scramble siRNA. After 24 h, CCN1 was detected by real-time PCR and western blot analysis. In addition, we selected the sequence with successful downregulation for further study. Sequences of the siRNA used for this analysis are the following: CCN1 siRNA, 5′-CCAGAAAUGUAUUGUUCAATT-3′ (forward [F]) and 5′-UUGAACAAUACAUUUCUGGTT-3′ (reverse [R]); scramble siRNA, 5′-UUCUCCGAACGUGUCACGUTT-3′ (F) and 5′-ACGUGACACGUUCGGAGAATT-3′(R).

### Statistical analysis

Data were represented as mean ± SEM of more than three separate experiments performed in triplicate. Statistical significance was compared between groups by the Student *t* test, after ANOVA analyses. *p* values less than 0.05 were considered to be significant.

## Results

**Fig. 1:**
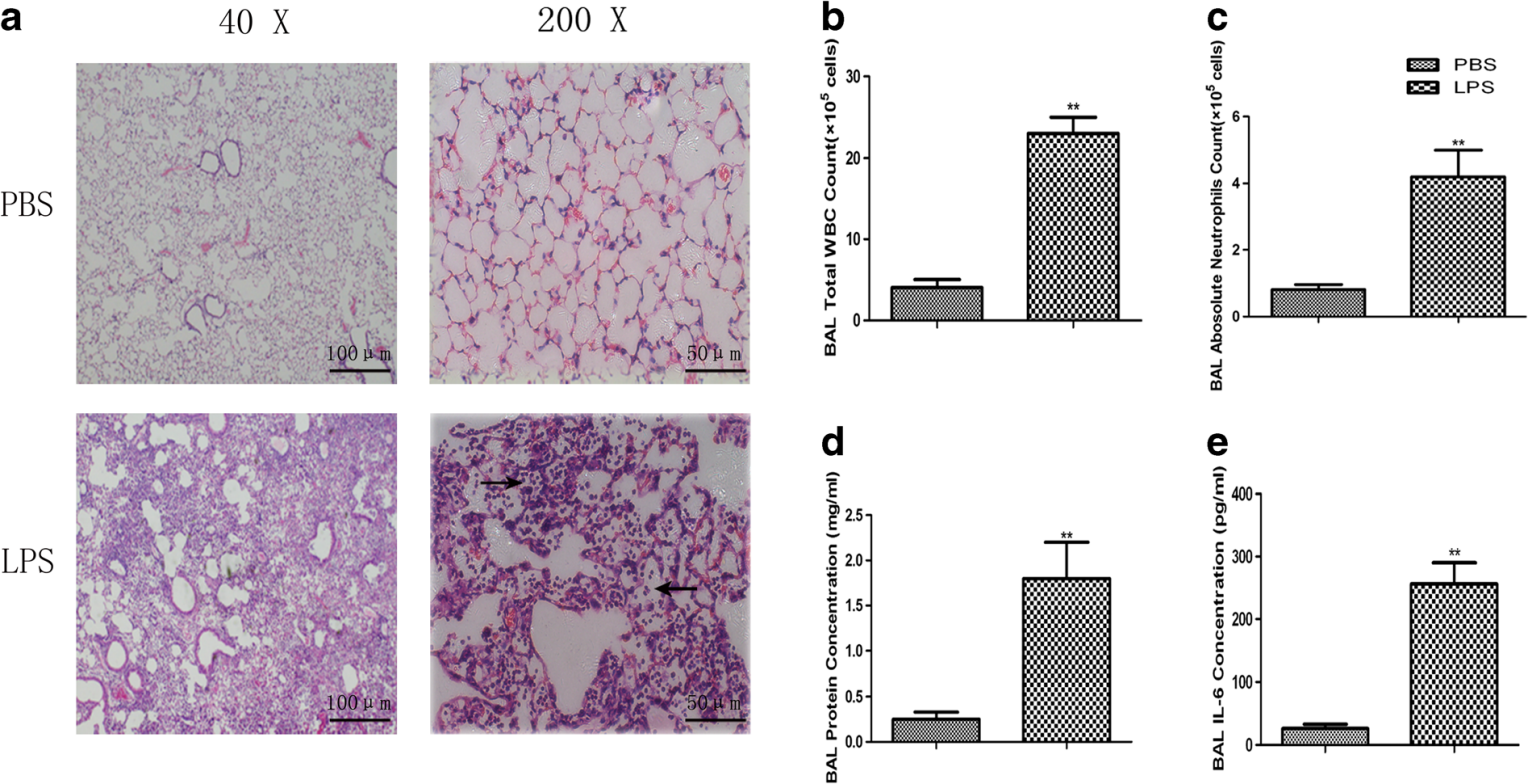
Effect of LPS on influx of inflammatory cells and mediator in endotoxin-induced ALI in mice. **a** The inflammatory cell influx, edema, blood, and thickening of the interstitium in lungs treated with LPS or PBS at 4 h. Hematoxylin staining (*blue*) indicates the localization of nuclei, and eosin staining (*red*) indicates the localization of the cytoplasm. Images were taken with a BX51 microscope DP71 camera at ×40 or ×200 magnification. The *arrows* point out the inflammatory cells. The number of total WBCs **b** and neutrophils **c** was counted by an Auto Hematology Analyzer, and the levels of total protein (**d**) and IL-6 (**e**) in BALF of ALI mice and controls were detected by ELISA kits. Data were presented as mean ± SEM of three independent experiments. ** stands for *p* values less than 0.01, as compared to controls (color figure online)

To evaluate pulmonary inflammation and endothelial barrier dysfunction, we detected leukocyte migration from the circulation into the alveolar space, and local and systematic production of inflammatory mediator IL-6. Our data demonstrated that most of the inflammatory cells were allocated within the interstitial area and the alveolar wall, and some in the alveolar space in animals pretreated with LPS, as compared with those in animals pretreated with PBS at 4 h (Fig. 1a). Consistent with these results, the number of total WBCs (Fig. 1b) and neutrophils (Fig. 1c) and the levels of total protein (Fig. 1d) and IL-6 (Fig. 1e) in BALF of ALI mice were significantly higher than those of the controls (*p* < 0.01).

**Fig. 2:**
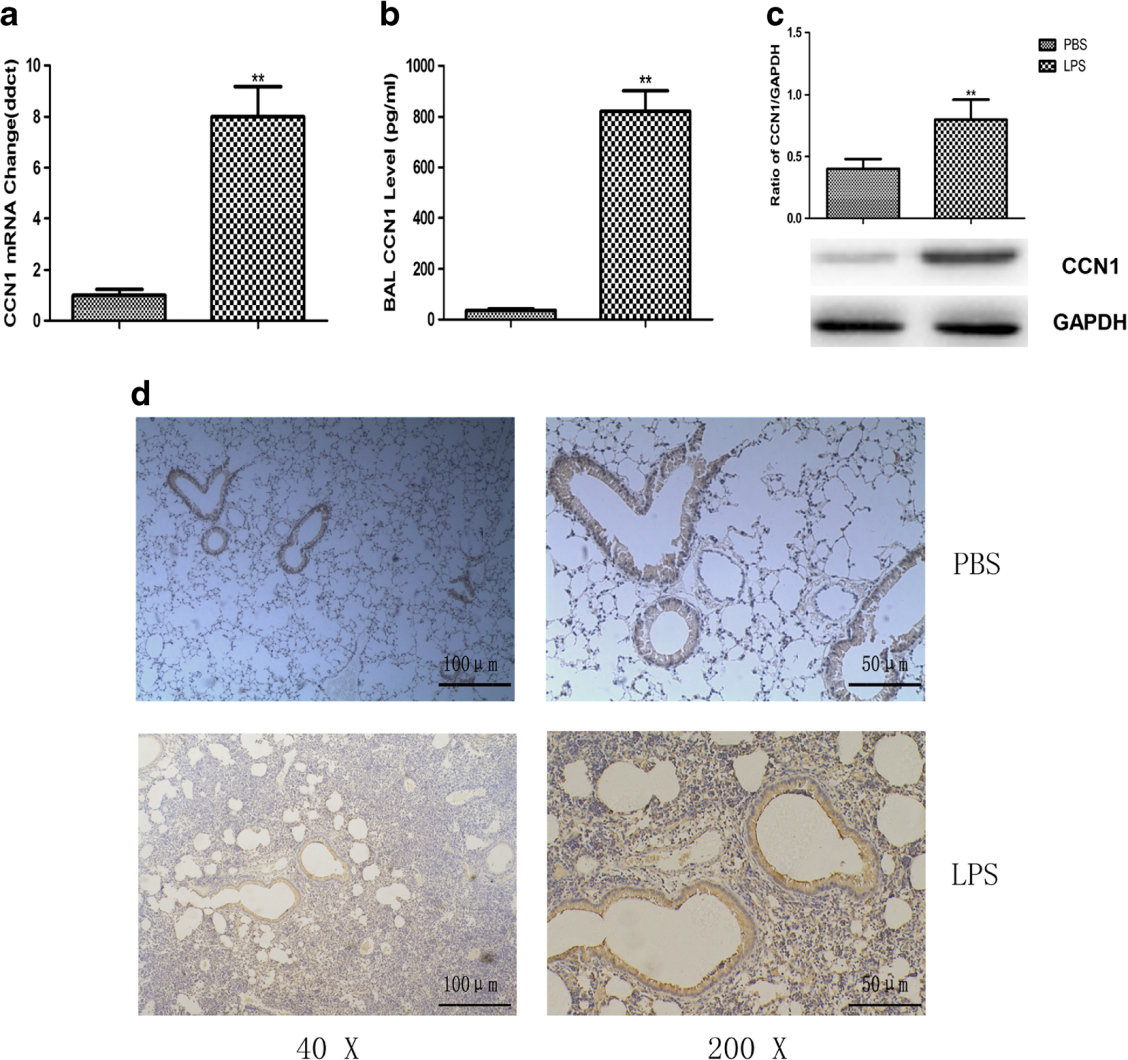
LPS induces increased expression of CCN1 in ALI mice. **a** The mRNA expression of CCN1 in lung tissues of mice treated with LPS or PBS. The CCN1 level in BALF (**b**) and protein expression (**c**) in lung tissues of mice treated with LPS or PBS. **d** The expression of CCN1 in lung tissues of mice treated with LPS or PBS was detected by immunohistochemistry analysis. Immunoreactivity of CCN1 mainly located around the bronchial epithelial cells in ALI mice. Data were presented as mean ± SEM of three independent experiments. ** stands for *p* values less than 0.01, as compared to the control

We examined gene and protein expression of CCN1 in mice intratracheally pretreated with LPS or PBS. As shown in Fig. 2, the messenger RNA (mRNA) expression of CCN1 in ALI mice was significantly increased upon LPS stimulus for 4 h (Fig. 2a). In a similar fashion, constitutive expression of CCN1 protein was found in the lung tissues and BALF of ALI mice (Fig. 2b, c). In addition, similar results were demonstrated by immunohistochemistry staining (Fig. 2d). These results indicated that high levels of CCN1 may be involved in the pulmonary inflammation of ALI mice.

**Fig. 3:**
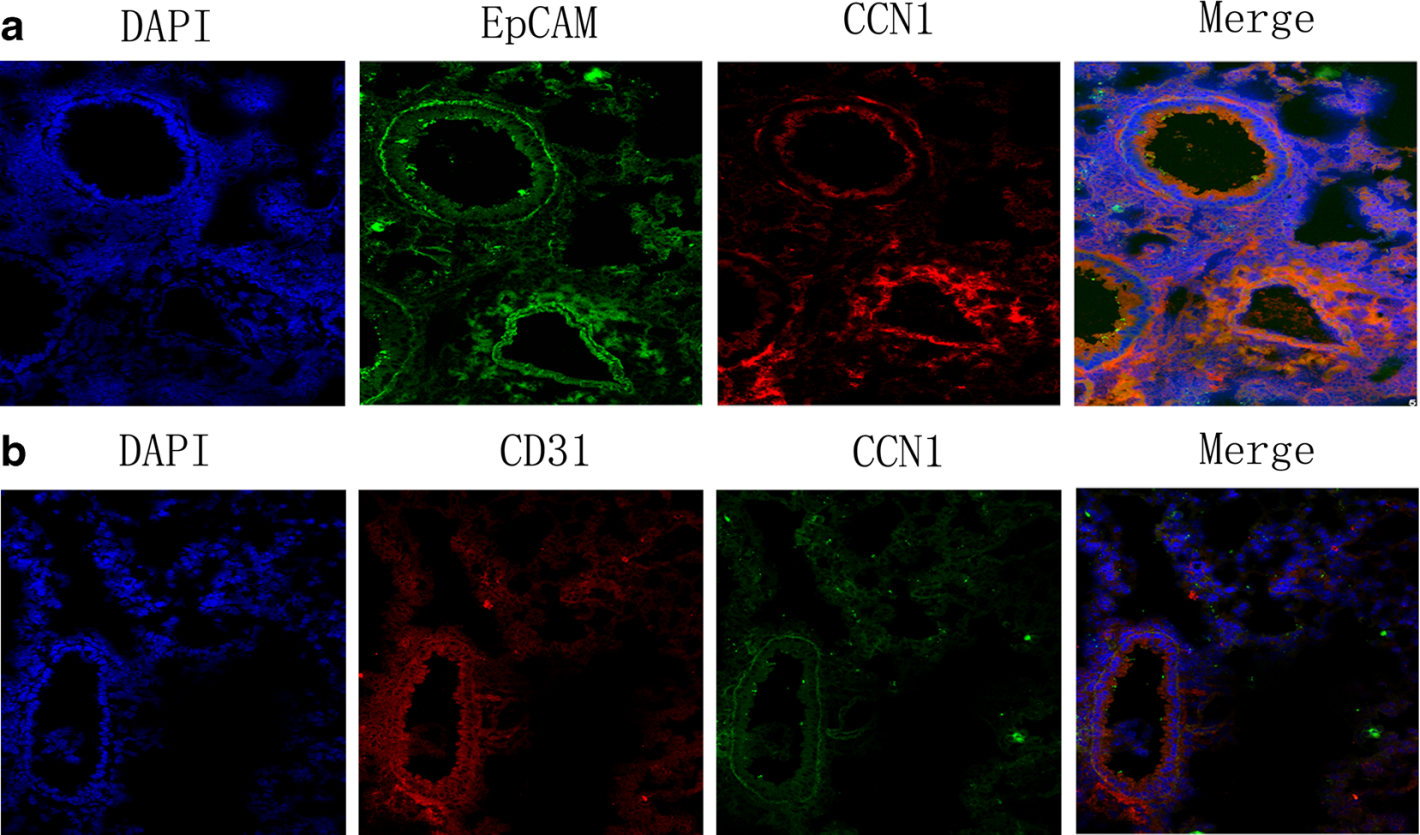
The localization of CCN1 in lung tissues of ALI mice. Immunofluorescence staining of EpCAM (**a**, *green*), CD31 (**b**, *red*), DAPI (*blue*), and CCN1 in mice challenged with LPS for 4 h (color figure online)

**Fig. 4:**
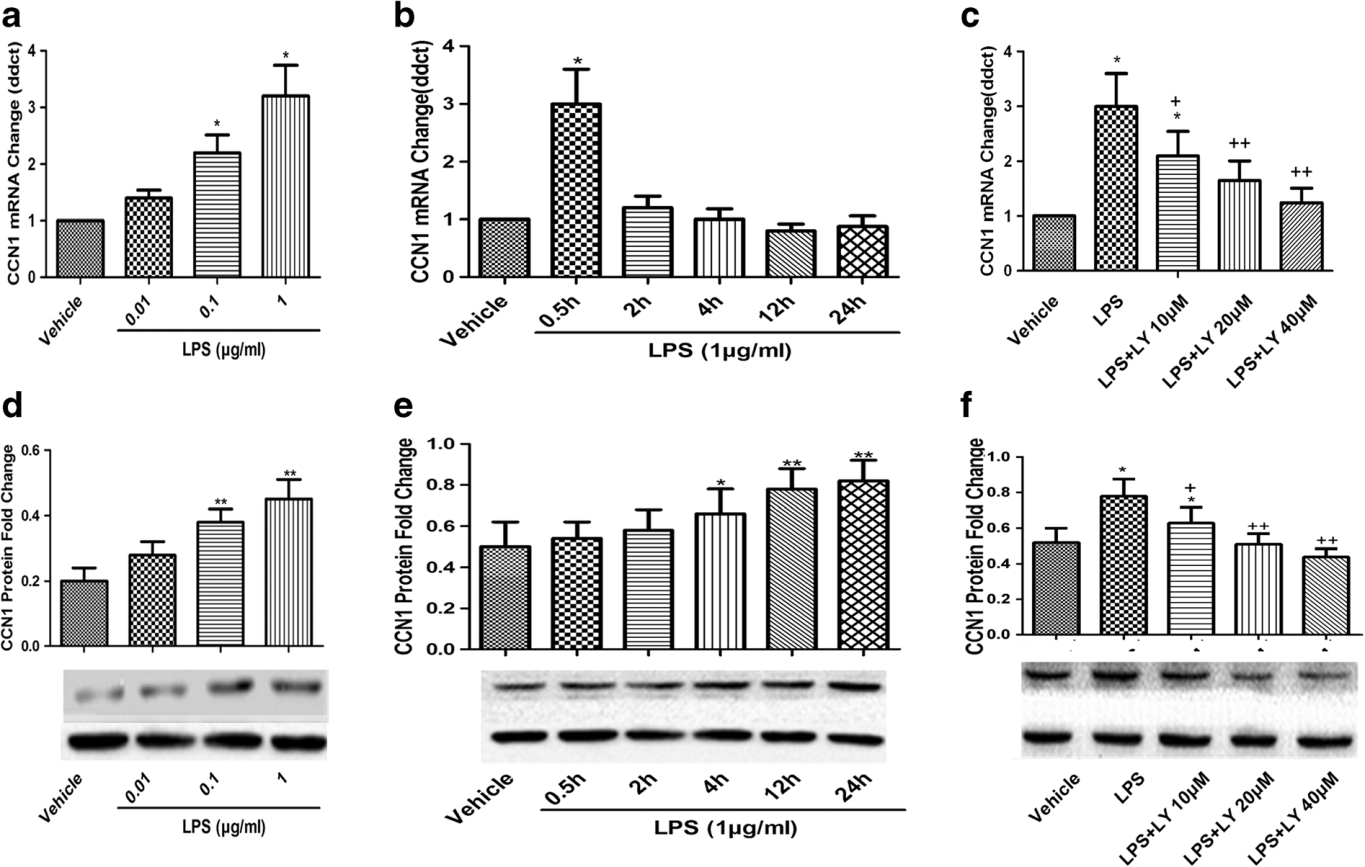
Involvement of the PI3K signal pathway in the regulation of CCN1 expression. **a** mRNA and protein (**d**) expression of CCN1 from 16HBE cells upon LPS stimulus (0.01, 0.1, and 1 μg/mL) was examined by real-time PCR at 0.5 h and western blot at 4 h. 16HBE cells were stimulated with LPS (1 μg/mL) for indicated times and examined by real-time PCR (**b**) and western blot (**e**) to detect mRNA and protein expression of CCN1 for indicated times. 16HBE cells were pretreated with DMSO and PI3K-specific inhibitor LY294002 (LY) at various concentrations for 2 h and then stimulated with 1 μg/mL LPS. Expression level of CCN1 was examined by real-time PCR at 0.5 h (**c**) and western blot at 4 h (**f**). Each data point represents mean ± SEM of three experiments. * and ** stand for *p* values less than 0.05 and 0.01, in comparison with DMSO alone, and + and ++ stand for *p* values less than 0.05 and 0.01, as compared to LPS (1 μg/mL), respectively

To further explore the specific cell involved in CCN1 production, epithelial-specific marker EpCAM and endothelial-cell specific marker CD31 and CCN1 were detected by immunofluorescence staining in lung tissues after LPS treatment. We found that CCN1 was mainly located around the bronchial epithelial cells (Fig. 3). In the in vitro studies, mRNA expression of CCN1 in 16HBE cells was greatly increased in a dose-dependent pattern and reached the peak level at 30 min after LPS stimulation (Fig. 4a, b). CCN1 protein was significantly increased after 4 h with LPS treatment (1 μg/mL), which was maintained from 4 h till 24 h (*p* < 0.05 or 0.01 in Fig. 4d, e, respectively). To investigate the signal pathway between LPS and CCN1, the PI3K-specific inhibitor LY294002 was used in the setting of LPS-induced CCN1 expression in 16HBE cells. As illustrated in Fig. 4c, f, gene and protein expression of CCN1 after LPS stimulation was significantly decreased by LY294002 treatment. These results demonstrated that LPS-induced PI3K activation was associated with significantly increased CCN1 expression in 16HBE cells.

**Fig. 5:**
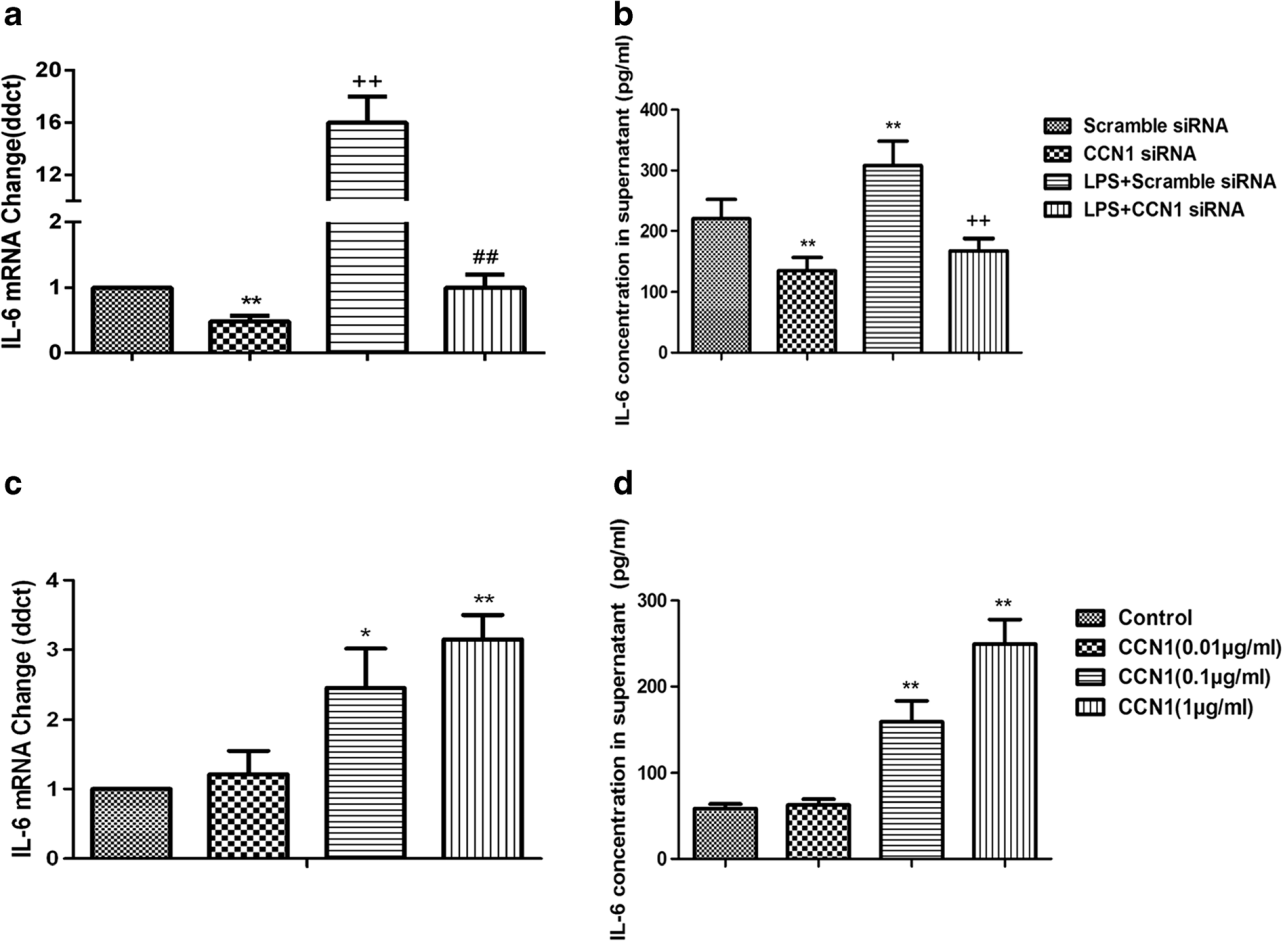
LPS-induced IL-6 production is CCN1-dependent in 16HBE cells. 16HBE cells were stimulated with LPS (1 μg/mL) after pretreatment with CCN1 siRNA and scramble siRNA. Total RNA was extracted and subjected to reverse transcription followed by real-time PCR to detect IL-6 mRNA at 4 h (**a**). IL-6 in cell-free supernatants after 24 h stimulated by LPS was assayed using ELISA (**b**). 16HBE cells were stimulated with CCN1 (0.01, 0.1, and 1 μg/mL) for 4 h to detect IL-6 mRNA (**c**) or 24 h to detect its secretion of IL-6 in supernatants (**d**). Each data point represents mean ± SEM of three experiments. ** stands for *p* values less than 0.01, as compared to scramble siRNA, and ++ stands for *p* values less than 0.01, as compared to LPS (1 μg/mL) and scramble siRNA, respectively

Consistent with the LPS-induced expression of CCN1, mRNA expression and protein production of IL-6 were significantly increased after stimulation of LPS at 1 μg/mL, as shown in Fig. 5a, b (*p* < 0.05 or 0.01, respectively). A positive correlation of CCN1 and IL-6 expression in 16HBE cells was observed. Cells were pretreated with CCN1 siRNA to investigate the potential role of endogenous CCN1 in LPS-induced over-expression and over-production of IL-6 mRNA and proteins. Pretreatment with CCN1 siRNA could significantly prevent the LPS-induced over-expression of IL-6 mRNA and over-production of IL-6 proteins, as compared with those pretreated with vehicle (*p* < 0.05 or 0.01 in Fig. 5a, b, respectively). Furthermore, recombinant human CCN1 from the dose of 0.1 to 1 μg/mL significantly increased the expression and production of IL-6 mRNA and proteins (*p* < 0.05 in Fig. 5c, d), as compared with those stimulated with vehicle. These observed effects were significantly inhibited when CCN1 was downregulated by the specific siRNA, confirming a partial role of CCN1 in LPS-induced IL-6 production.

## Discussion

Inflammatory cascade reaction has been recognized as a central stage in the pathophysiology of ALI/ARDS, characterized by infiltration of neutrophils, lymphocytes, and macrophages (Maron-Gutierrez et al. 2014; Rafat et al. 2013; Sharp et al. 2015; Zhou et al. 2012). Besides these bone marrow-derived inflammatory cells, the structural airway epithelial cells are involved as well. Accumulating evidence indicates that the airway epithelial cells play important roles in not only initiating local and systemic inflammation but also activating production of pro-inflammatory cytokines/chemokines (Jia et al. 2016; Wang et al. 2007). Among all the cytokines and chemokines, robustly increased IL-6 levels were found in plasma and BALF of ALI/ARDS patients and mice (Chen et al. 2016; Jia et al. 2016). The upregulation and secretion of IL-6 were regulated transcriptionally by several transcription factors such as NK-κB, Lin28, and Let-7 and involved in different inflammation and immune responses (Iliopoulos et al. 2009; Sung et al. 2013). The present study provided additional evidence that the number of total WBCs and neutrophils and the levels of IL-6 in BALF of ALI mice were significantly higher than those of the controls, and identified a novel ECM protein CCN1 whose levels correlated with the IL-6 production. These findings, consistent with our observations from the previous study, prompted us to hypothesize that CCN1 may be critically involved in IL-6 secretion, further accelerating the development of the local inflammatory microenvironment.

CCN1 is highly expressed in inflammation and injury repair and regulates varied and divergent cellular responses in a cell type- and context-dependent manner (Lau 2011). Recent reports demonstrated that CCN1 could ameliorate skin (Jun and Lau 2010) and liver fibrosis (Kim et al. 2013), while promoting pro-fibrotic responses in the kidney (Lai et al. 2014). Studies of the role of CCN1 in the lung have not been completely explored, although over-expression of CCN1 mediated neutrophilic alveolitis and acute lung injury in mice (Grazioli et al. 2015). Our study found that CCN1 was mainly located around the bronchial epithelial cells in ALI mice, and we illustrated a novel communicator CCN1 connecting the initial noxious stimulation (LPS) to the end effectors (IL-6) in bronchial epithelial cells. In contrast to the elegant studies by Jun and Lau (2010) and Kim et al. (2013), the present results support a different role for CCN1 in tissue injury repair, which implied that the development of a conceptual framework on how this CCN1 functions in inflammation and injury repair should take into account tissue- or cell-specific factors and the specific models utilized.

Previous studies indicated that many regulatory factors may involve in process that LPS induces secretion of inflammatory mediators in bronchial epithelial cells (Moon et al. 2013). Results from the present study demonstrated that both endogenous and exogenous CCN1 could induce IL-6 production. And the induced levels of IL-6 were blocked by CCN1 siRNA, indicating IL-6 induction was largely CCN1-dependent. This finding might suggest a potential mechanism that CCN1 acts a central molecule bridging vicious stimuli to pro-inflammatory cytokines. Furthermore, CCN1, as a secreted molecule, connected bronchial epithelial cells to the matrix. By modulating the release of IL-6, it further provided a cross-talk between epithelial cells and lung inflammatory cells. Combined with the previous studies that CCN1 secretion induced by cigarette smoking extracts augments IL-8 release from bronchial epithelial cells (Moon et al. 2013), our current report further extended the importance of CCN1 in triggering lung inflammation.

Consistent with the previous finding on the role of the PI3K signal pathway in lung inflammation and remolding through regulating activation of epithelial cells and transdifferentiation of fibrogenic cells (Chen et al. 2011; Fang et al. 2013), we found that the PI3K signal pathway may play an essential role in LPS-induced CCN1 production from bronchial epithelial cells, evident with the finding that the induction of CCN1 by LPS was partially prevented by a PI3K inhibitor. These results implied that the LPS-PI3K-CCN1-IL-6 axis might be a new therapeutic target of inflammatory in ALI/ARDS.

**Fig. 6:**
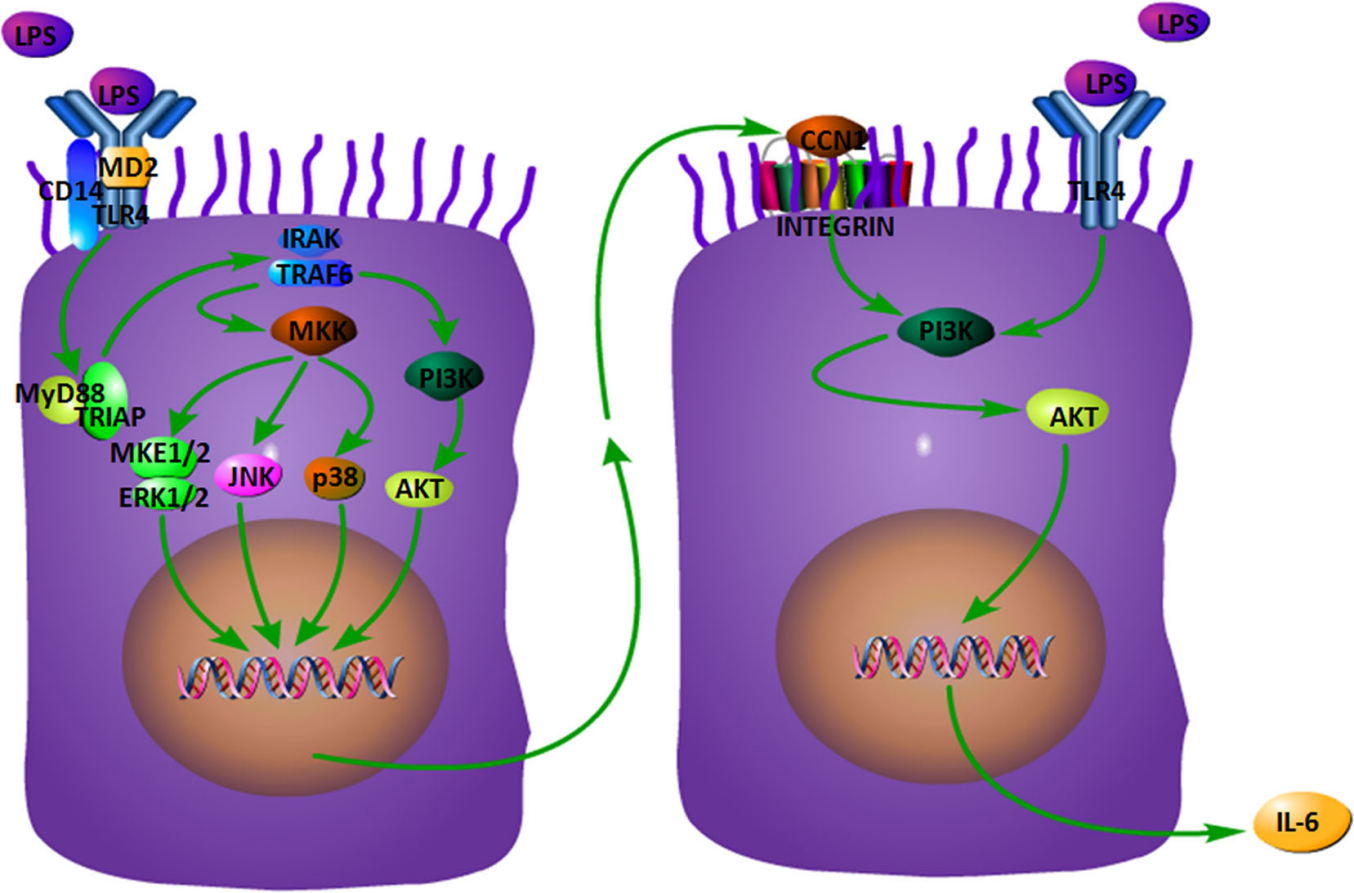
Proposed mechanism of CCN1 production and function in airway epithelial cells. Airway epithelial cells per se act as the initiator, receptor, or promoter of CCN1 responsible for the IL-6 production and formation of inflammatory microenvironment during ALI/ARDS

There are also some limitations in the present study: the bronchial epithelial cell 16HBE employed in this study is a HPV-16 E6/E7-transformed, immortalized bronchial epithelial cell, and more bronchial epithelial cell lines and primary cells from mouse should be used. In addition, more pro-inflammatory mediators relevant to ALI/ARDS except IL-6 need to be evaluated in the future. Future directions on the role of CCN1 in ALI/ARDS in vivo include the following: (1) examining the levels of CCN1 and IL-6 in BALF of CCN1 transgenic mouse; (2) administration of a bioactive PI3K inhibitor in vivo and examining the IL-6 secretion in BALF, in the presence and absence of LPS stimulation. In summary, the CCN1 expression and secretion from bronchial epithelial cells are robustly increased in the inflammatory condition and/or stimuli like LPS. This elevated matrix CCN1 plays a crucial role in IL-6 release, which can be blocked by the PI3K inhibitor (Fig. 6). As a communicator between lung parenchymal cells and inflammatory cells, as well as a connector between intracellular components and ECM, CCN1 might be a potential cellular therapeutic target for ALI/ARDS.

## Electronic supplementary material

Supplementary Figure 1 Effect of PI3K inhibitor LY294002 on CCN1 production. The mRNA and protein expression of CCN1 in 16HBE cells after LY294002 stimulation. (GIF 18 kb)

High resolution image (TIFF 567 kb)
