# Supplementary figures and images for: Lipopolysaccharide-induced CCN1 production enhances interleukin-6 secretion in bronchial epithelial cells
Source: Cell Biol Toxicol. 2017 Jun 21;34(1):39–49. doi: 10.1007/s10565-017-9401-1 (PMC5775366; doi:10.1007/s10565-017-9401-1)

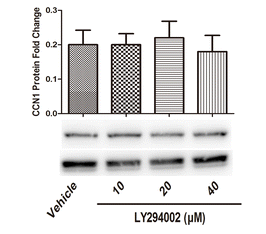

Supplement: Supplementary file 1 — Effect of PI3K inhibitor LY294002 on CCN1 production. The mRNA and protein expression of CCN1 in 16HBE cells after LY294002 stimulation. (GIF 18 kb) [file 10565_2017_9401_Fig7_ESM.gif]

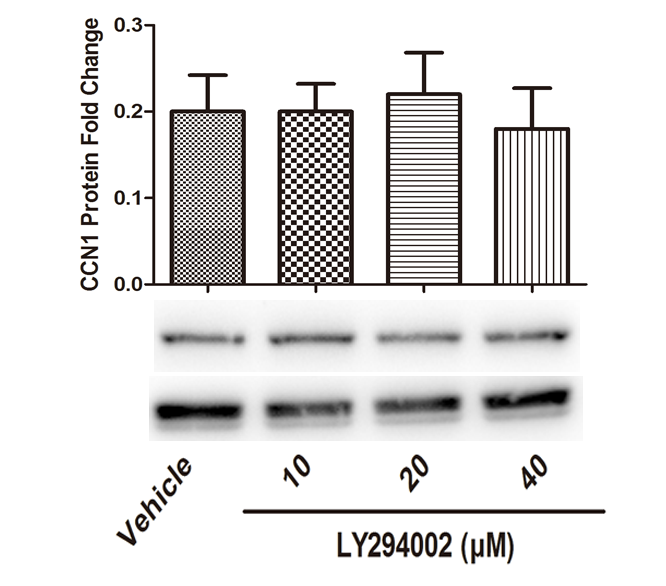

Supplement: Supplementary file 2 — High resolution image (TIFF 567 kb) [file 10565_2017_9401_MOESM1_ESM.tif]
